# Dealing With Discrimination, Harassment and Bullying in Mental Health Settings: A Qualitative Evaluation of Training

**DOI:** 10.1192/bjo.2024.293

**Published:** 2024-08-01

**Authors:** Lachlan Fotheringham, Katie Munro

**Affiliations:** 1 Cumbria, Northumberland, Tyne and Wear NHS Foundation Trust, Newcastle Upon Tyne, United Kingdom; 2 Newcastle University, Newcastle Upon Tyne, United Kingdom

## Abstract

**Aims:**

This is a qualitative evaluation of a simulation/debrief based training session to address discrimination in an NHS workplace, delivered to psychiatry trainees. Videos portraying discrimination acted as the simulation, followed by a diamond model informed debrief.

This evaluation aimed to:

- assess the effectiveness of this training session in terms raising awareness of discrimination, problematising discrimination, empowerment to act and skills building
- explore the extent to which these stated aims are relevant to participants’ experience of discrimination, harassment and bullying at work
- establish if this is a meaningful and acceptable training model for this topic
- establish if there are more relevant themes that this training session should be focusing on and if so, what these are?

**Methods:**

A total of 8 trainees were interviewed between December 2022 and May 2023, having recently completed the training. A thematic analysis was undertaken by two researchers following established recommendations, seeking to bring out latent themes with an inductive, interpretative approach within a constructionist paradigm.

**Results:**

Trainees attended with existing knowledge, skills and attitudes about discrimination, harassment and bullying, and about the training session itself.

Both the simulation and debrief were valued by trainees. The debrief was more than just a discussion. Portrayals of discrimination in the videos/simulation could have been more subtle, and tackled a more diverse range of examples such as LGBTQ+.

The learning objectives were largely met, and related to real challenges that trainees face. Trainees took away more than this, citing learning related to team cohesion and developing their sense in which discrimination in the context of mental illness requires special consideration.

**Conclusion:**

This model of training is providing good value in addressing a topic of strategic importance in a novel way. The impact on empowerment and skills development is likely to be particularly valuable in impacting real world responses to workplace experiences of discrimination. Promoting team cohesion and a space to thoughtfully consider the special case of discrimination in the context of mental illness are important additional benefits. The simulation/debrief model is likely to be crucial, providing learning which would be inaccessible to didactic or e-learning based modes of delivery. The simulation materials may be improved by depicting LGBTQ+ issues, and a more subtle portrayal of discrimination. While this evaluation was situated in a psychiatric context, it could have wide applicability to tackling similar challenges throughout NHS workplaces.

